# Implementation of case management to reduce cardiovascular disease risk in the Stanford and San Mateo Heart to Heart randomized controlled trial: study protocol and baseline characteristics

**DOI:** 10.1186/1748-5908-1-21

**Published:** 2006-09-27

**Authors:** Jun Ma, Ky-Van Lee, Kathy Berra, Randall S Stafford

**Affiliations:** 1 Program on Prevention Outcomes and Practices, Stanford Prevention Research Center, Stanford University School of Medicine, Stanford, CA, USA

## Abstract

**Background:**

Case management has emerged as a promising alternative approach to supplement traditional one-on-one sessions between patients and doctors for improving the quality of care in chronic diseases such as coronary heart disease (CHD). However, data are lacking in terms of its efficacy and cost-effectiveness when implemented in ethnic and low-income populations.

**Methods:**

The Stanford and San Mateo Heart to Heart (HTH) project is a randomized controlled clinical trial designed to rigorously evaluate the efficacy and cost-effectiveness of a multi-risk cardiovascular case management program in low-income, primarily ethnic minority patients served by a local county health care system in California. Randomization occurred at the patient level. The primary outcome measure is the absolute CHD risk over 10 years. Secondary outcome measures include adherence to guidelines on CHD prevention practice. We documented the study design, methodology, and baseline sociodemographic, clinical and lifestyle characteristics of 419 participants.

**Results:**

We achieved equal distributions of the sociodemographic, biophysical and lifestyle characteristics between the two randomization groups. HTH participants had a mean age of 56 years, 63% were Latinos/Hispanics, 65% female, 61% less educated, and 62% were not employed. Twenty percent of participants reported having a prior cardiovascular event. 10-year CHD risk averaged 18% in men and 13% in women despite a modest low-density lipoprotein cholesterol level and a high on-treatment percentage at baseline. Sixty-three percent of participants were diagnosed with diabetes and an additional 22% had metabolic syndrome. In addition, many participants had depressed high-density lipoprotein (HDL) cholesterol levels and elevated values of total cholesterol-to-HDL ratio, triglycerides, triglyceride-to-HDL ratio, and blood pressure. Furthermore, nearly 70% of participants were obese, 45% had a family history of CHD or stroke, and 16% were current smokers.

**Conclusion:**

We have recruited an ethnically diverse, low-income cohort in which to implement a case management approach and test its efficacy and cost-effectiveness. HTH will advance the scientific understanding of better strategies for CHD prevention among these priority subpopulations and aid in guiding future practice that will reduce health disparities.

## Background

Coronary heart disease (CHD) affects 13 million Americans and is estimated to have cost the US 142 billion dollars in 2005 [1]. The current primary care delivery model lacks a multidisciplinary infrastructure that is conducive to effective management of multiple CHD risk factors [2]. The growing strain of chronic disease management on the health care system leaves physicians little time for preventive care [3], which paradoxically is indispensable for the treatment and prevention of many chronic diseases including CHD. While CHD affects every racial/ethnic group and social class, ethnic minorities and persons of low socioeconomic status (SES) disproportionately bear the burden of CHD and its major risk factors [1,4], These population subgroups also are more likely to receive substandard cardiac care compared with whites and individuals of higher SES [5]. Accelerating the translation of research into community-based practice and enhancing health impact in disparate populations have been identified as strategic imperatives for the elimination of inequities in cardiovascular health [6].

Alternative approaches are needed to supplement traditional one-on-one sessions between patients and doctors. One such approach is integrated care delivered through case management (CM). Case management is a comprehensive, longitudinal approach that involves a multidisciplinary team of health care providers, such as physicians, nurses and dietitians, who simultaneously intervene to reduce multiple risk factors for a disease, such as CHD. Randomized clinical trials have established the efficacy of intensive case management intervention to reduce multiple cardiovascular risk factors among predominantly white, high-risk patients [7-10]. Only recently has this therapeutic approach been tested among ethnic minorities, where it was found to be effective [11]. Researchers have urged greater implementation of case management [12].

Chronic disease management for ethnic minorities of low-SES represents unique and difficult challenges for local health care systems, many of which are overburdened by a complex clinical load and have a primary care delivery model that is not well designed to provide intensive chronic disease management.

The Stanford and San Mateo Heart to Heart (HTH) project is designed to conduct a randomized controlled clinical trial that rigorously evaluates the efficacy and cost-effectiveness of a case management intervention in reducing cardiovascular risk among patients of the San Mateo County Medical Center (SMMC) in California, U.S.A. Based on outcomes data available through the clinical trial, HTH staff will then facilitate implementation of the HTH case management model as an ongoing disease management program within SMMC. This report details the study design, methodology, and baseline sociodemographic, clinical and lifestyle characteristics of 419 randomized participants. We expect participants to have sociodemographic characteristics that differ significantly from those of the San Mateo County and U.S. adult populations. We also anticipate that participants will possess clinical and lifestyle risk factors that predict elevated risk of future cardiovascular events and that these risk factors can be modified through intense medical and/or lifestyle interventions.

## Methods

The study was approved by the Stanford Institutional Review Board (IRB) and an independent IRB responsible for reviewing study protocols for the San Mateo Medical Center (SMMC).

### Study setting

San Mateo County in California is a study in contrasts – although this mostly suburban county includes some of the most expensive housing in the nation, it has a sizable population of lower-SES persons with demographic characteristics comparable to urban areas. As of 2004, the racial composition of San Mateo County was 62% White, 26% Asian/Pacific Islander, and 4% Black. Twenty-two percent of the population self-identified as Hispanic and 32% as foreign-born [13]. Within San Mateo County, heart disease is the leading cause of death (29% of all deaths during 1997–2001) while stroke is third [14]. In 2004, 7% of the adult population had diabetes with the highest prevalence (15%) among persons aged 65 and older. In addition, 86% of San Mateo County adult residents had reported at least one cardiovascular risk factor, *e.g.*, 75% for overweight and obesity, 55% for physical inactivity, 26% for hypertension, 25% for hyperlipidemia, and 12% for smoking. As a branch of the county government, the SMMC serves a significant portion of the county population that has low SES and lacks private health insurance. The SMMC has approximately 106 physicians per 100,000 people, whereas the national average was 289/100,000 in 2000.

### Study design

HTH is a 5-year project that consists of a randomized controlled clinical trial in the first 4 years and a transition phase in the last year. The 2-armed clinical trial (Immediate vs. Delayed Intervention) was designed to enroll 400 patients. In an intention-to-treat analysis, this sample size yields 87% power to detect a mean change of 5 points in the Framingham risk score, with an SD of 10, at an α level of 0.01 after accounting for a 25% loss to follow-up.

Participants in both intervention groups continue to receive usual medical care throughout the study period. In addition, participants randomized to Immediate Intervention receive intensive case management for CHD risk reduction for 15 months and then a maintenance program for a minimum of 12 months to assess the durability of initial intervention changes. Participants randomized to Delayed Intervention serve as control for Immediate Intervention patients for the first 15 months and then receive intensive case management for 15 months. The switching-over design not only addresses ethical concerns about withholding treatment from half the study sample, but will also enable us to assess whether the intervention had equal impact whether provided to a naïve population or to a group followed in usual care for 15 months. We will compare change in CHD risk from baseline to 15 months for Immediate Intervention with that from 15 months to 30 months for Delayed Intervention. Similar magnitudes of change in CHD risk between the two study arms would imply that the Delayed Intervention arm was not notably contaminated by the intervention and measurement process and that no noteworthy differences were caused by the 15-month difference in time *per se*.

Immediate Intervention patients who complete their 12-month maintenance period and Delayed Intervention patients who complete their 15-month case management period remain under maintenance case management until they are fully transitioned back to the care of the SMMC in the last year of the project. In addition to patient transition, we will also transition the HTH case management model, as guided by outcomes data from the clinical trial, into an ongoing disease management program operated by SMMC. By including this transition phase, we will be able to assure continuity in patient care and also test the feasibility and effectiveness of implementing our intervention in a community practice setting.

### Recruitment

Between October 2003 and April 2005, 1005 patients were referred by physicians at four SMMC outpatient clinics located in Menlo Park, Redwood City, South San Francisco, and Daly City. These four clinics were chosen for geographic proximity, accommodating clinic environment, sufficient patient volume, diverse patient demographics, and established adult primary care services. All data acquisition and case management visits took place at the clinic where the patient usually receives his/her primary care.

**Figure 1:**
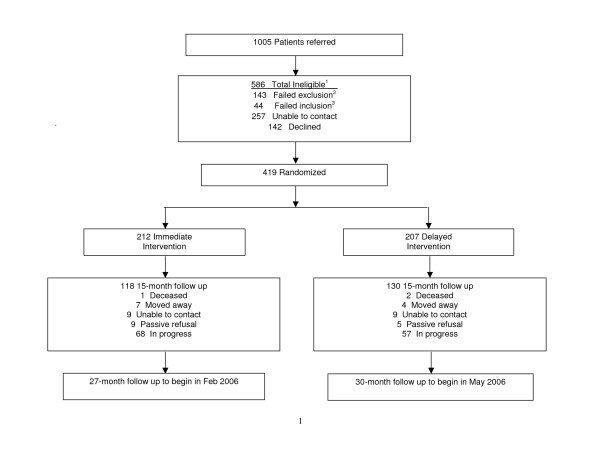
Enrollment and follow-up in the Stanford and San Mateo County Heart to Heart Trial. ^1^A patient may be ineligible for more thanone reason. ^2^Number of participants who failed to meet exclusion criteria: being resident in long-term facility (n = 1); moving away soon (26); age ≤ 35 or ≥ 85 (13); significant comorbidities (10); substance abuse (2); no telephone (1); family member already enrolled (7); anticipated absence >4 months (18); difficulty coming to appointments (35); participating in other research programs (21); pregnant or planning to become pregnant (2); no English or Spanish and no interpreter (7). ^3^Number of participants who failed to meet inclusion criteria: Has CAD or CAD risk equivalent but did not have any of the CHD risk factors specified in Table 1 (n = 2); does not have CAD or CAD risk equivalent and did not have any of the CHD risk factors specified in Table 1 (n = 42).

Physicians at the study clinics were instructed to refer male and female patients between the ages of 35 and 85 who had CHD, CHD risk equivalents (*i.e.*, abdominal aortic aneurysm, peripheral vascular disease, carotid artery disease, or diabetes mellitus), or moderately to severely elevated levels of major CHD risk factors. We were unable to contact 257 of the 1005 referred patients and an additional 142 declined participation (Figure 1). We screened the remaining 596 patients by phone or at the baseline visit and excluded 143 patients for failing to meet the exclusion criteria. These criteria identify patients with circumstances that may severely limit their ability complete the study protocol or that may confound results of the study. The footnotes in table 1 list percentages by exclusion criteria. During the same phone call, those not excluded were scheduled for a baseline visit. Table 1 enumerates study inclusion and exclusion criteria. Consent forms were available in English and Spanish and the patient's informed consent was obtained before the start of the baseline visit. For patients who spoke a language other than English or Spanish, a family member over the age of 18 or a SMMC staff member served as interpreter. As part of the baseline evaluation, biophysical measures were obtained that further excluded 44 patients not meeting inclusion criteria.

**Table 1:** Inclusion and exclusion criteria.

**Inclusion criteria**
The patient has CAD or CAD risk equivalent (abdominal aortic aneurysm, peripheral vascular disease, transient ischemic attack, stroke, diabetes, or FBS ≥ 126 mg/dL × 2) and has at least one of following: SBP ≥ 130 mmHg, DBP ≥ 80 mmHg, LDL ≥ 100 mg/dL, HDL ≤ 40 mg/dL, TG ≥ 150 mg/dL, FBS ≥ 126 mg/dL, BMI ≥ 30, or is a current smoker.
The patient does not have CAD or CAD risk equivalent but has at least one of the following: SBP ≥ 160 mmHg, DBP ≥ 100 mmHg, LDL ≥ 190 mg/dL, TC ≥ 240 mg/dL, TG ≥ 500 mg/dL, HbA1c ≥ 8.0%, BMI ≥ 35, or is a current smoker.
The patient does not have CAD or CAD risk equivalent but has at least two of the following: a) SBP ≥ 140 mmHg or DBP ≤ 90 mmHg, b) HDL ≤ 40 mm/dL or TG ≥ 200 mg/dL, c) LDL ≥ 160 mg/dL or TC ≥ 240 mg/dL, d) FBS ≥ 110 mg/dL × 2, or e) male age ≥ 45 or female age ≥ 55 or with positive family history of CAD.

Abbreviations: FBS = fasting blood sugar, SBP = systolic blood pressure, DBP = diastolic blood pressure, LDL = low-density lipoprotein, HDL = high-density lipoprotein, BMI = body mass index, TC = total cholesterol, TG = triglycerides, HbA1c = hemoglobin A1c.

**Exclusion criteria**
Resident of long-term facility.
Lack of spoken English or Spanish by patient or household member ≥ 18 years old who can serve as interpreter.
Moving before end of intervention (30 months).
Age ≤ 35 or ≥ 85.
Significant comorbidities such as: uncontrolled metabolic disorders (renal failure, liver failure, etc.), active symptoms suggesting acute myocardial infarction or decompensated congestive heart failure, Malignancy or other condition limiting life expectancy, psychiatric disorder with active manifestations.
Substance abuse.
No telephone or means of contacting patient.
Family household member already enrolled.
Homeless and not living with relatives/friends.
Anticipated absence for more than 4 consecutive months.
Difficulty coming to appointments approximately every 1–2 months.
Already participating in the Diabetes program.
Currently pregnant or intends to get pregnant the next 3 years.

### Randomization

A total of 419 patients met all study criteria and provided informed consent. They were randomized into Immediate or Delayed Intervention groups, using the permuted block method stratified by gender and ethnicity (Hispanic vs. Non-Hispanic). A statistician independent of the study generated a sequence of 100 randomization IDs and treatment assignments per clinic for each of the four combinations of gender and ethnicity, *i.e. *1) female, Hispanic, 2) male, Hispanic, 3) female, non-Hispanic, and 4) male, non-Hispanic. An administrative assistant who is not involved in the study printed the IDs and corresponding treatment assignments on separate pages and sealed each page into an opaque envelope. The administrative assistant then placed these envelopes by stratification group and in randomization sequence into four envelope containers for use at each clinic. All case managers were masked to randomization sequence and treatment assignments. At the baseline/randomization visit, each eligible and willing participant was instructed to take the envelope in the very front of the appropriate container, open the envelope in the presence of the case manager, and read his group randomization. The participant would then sign the randomization form and the case manager would record the participant's randomization assignment and the randomization ID number on the randomization disposition form.

Randomization occurred at the patient level in this trial. Randomization at the clinic or physician level would cast detrimental doubts on internal validity of the trial as it would not be feasible to guarantee a balanced distribution of the diversity of clinic sites and physician practice patterns across the study arms. Consequently, it would be difficult to determine whether outcomes reflected the intervention or differences in patient populations across sites and differences in physician practice patterns. A drawback of patient-level randomization is the possibility of contamination. We expect the extent and impact of contamination to be modest, however. By design, study-specific case managers who are independent of existing physician practices within the study clinic sites provide case-management to the intervention patients and the scheduling process is separate from that of the clinical sites. Much of the value of the intervention comes from activities that are not usually given high priority by primary care physicians. In addition, any potential for contamination produces a conservative bias, reducing the measured impact of the intervention and biasing the findings towards the null hypothesis. Furthermore, our statistical methods will specifically address the degree of intra-class correlation within physician's practices and thereby assess the likelihood and potential extent of contamination.

### Study measurements

The primary outcome measure is the absolute CHD risk over 10 years. For participants without known CHD, the Framingham risk assessment algorithms published by Wilson et al. [33] will be used to estimate the 10-year risk probability of CHD on the basis of sex, age, systolic blood pressure, total cholesterol (TC), cigarette smoking status, and diabetes status. For participants with existing CHD, their 10-year CHD risk will be extrapolated from 2-year probability estimates [34] after accounting for aging effect and censoring individuals with new-onset CHD as time elapses. Secondary guideline-based outcome measures include low-density lipoprotein cholesterol (LDL-C), high-density lipoprotein cholesterol (HDL-C), systolic and diastolic blood pressure, hemoglobin A1c (HbA1c), physical activity, smoking status, body mass index (BMI), dietary intake of total and saturated fat and fruits and vegetables, use of recommended medications (*e.g.*, aspirin, statins, thiazide diuretics, beta-blockers, and angiotensin-converting enzyme inhibitors [ACEIs]/angiotensin receptor blockers [ARBs]).

Outcome measurements are designed to occur at baseline and 15 months for all patients, at 27 months for Immediate Intervention, and at 30 months for Delayed Intervention patients. HTH case managers, including one nurse practitioner, two registered nurses, and two registered dietitians, completed all baseline measurements, which consisted of biophysical measurements, and lifestyle, social, and demographic questionnaires. Baseline visits were conducted in one or two clinic visits, depending upon patient and case manager schedules.

Height was measured by a wall stadiometer and weight by a digital balance scale. Both of these measures were taken without shoes and while wearing light clothing. BMI was then calculated (in kg/m^2^). Waist circumference (in cm) was measured in standing position using a cloth tape measure placed at the level of the iliac crest. Blood pressure was measured in both arms, using the brachial artery, after 10 minutes of sitting in a relaxed position, and the average of two readings was used. After ascertaining that the patient had fasted for ≥ 12 hours, a finger stick blood sample was obtained for measurement of plasma TC, HDL-C, LDL-C, triglyceride (TG), and glucose levels using the Cholestech LDX point of service testing system (Cholestech Corporation, Hayward, CA). Plasma HbA1c was obtained utilizing the Cholestech GDX.

Patients were asked about medical and family history related to cardiovascular disease (CVD). They were asked to bring all medications, including supplements and nutraceuticals, with them to the baseline visit. Medication name, medication class, dosage, and therapeutic purpose were recorded. Patients with CHD and/or diabetes were specifically asked about their use of β-blockers, statins, ACEIs/ARBs, and aspirin. Health care utilization was assessed by asking about hospitalizations, emergency room visits, and outpatient visits within the past six months.

Case managers recorded patient age, gender, ethnicity (*i.e. *Latinos/Hispanics vs. others including traditional racial categories), education, marital status, employment status, and household size. Standardized questionnaires were administered to collect data on cigarette smoking (short form of stage of change [15]), nicotine dependence for current smokers (Fagerstrom Tolerance Questionnaire [16]), self-perceived health-related quality of life (SF-12 Health Survey [17]), self-reported depression (CES-D scale [18]), fruit, vegetable and fat intake (Block screeners [19,20]), and physical activity (Stanford 7-Day Recall [21]).

All baseline measurements are repeated at two follow-up visits: a first follow-up planned for 15 months and a second planned for 27–30 months. The 15-month follow-up measurements are expected to be completed by August 2006 and the 27–30-month follow-up measurements by October 2007. During follow-up evaluations, case managers continue to collect all biophysical measurements; however, questionnaires are administered by trained research assistants who are masked to treatment assignments. Research assistants also are responsible for calling patients at 7 months and 22 months to obtain interim data on health care utilization.

### Intervention

HTH intervention protocols specifically focused on CHD risk reduction. Non-CHD-related conditions remained the responsibility of the patient's PCP, although we often facilitated having the primary care provider address specific patient needs.

#### Immediate intervention

HTH case-management intervention was based on the latest guidelines for the management of CHD risk factors, particularly those reflecting cholesterol management [22,23], hypertension [24], physical activity [25], diabetes [26], aspirin therapy [27,28], smoking [29], obesity management [30], and primary and secondary CHD prevention [31,32]. Specific lifestyle and medical protocols for case managers were developed from these guidelines and continue to be updated based on new evidence. Each patient was managed by a nurse practitioner/registered nurse and a dietitian. Guided by the intervention protocols, the intensity of case management was individualized based on patient risk profile, patient preferences, and available resources within the community. The aim for each patient was to improve individual risk factors and reach recommended goals. Supervised by two physicians and a senior nurse practitioner, the case managers reviewed, adjusted as necessary, and monitored medical therapies in accordance to guidelines and the SMMC formulary. Lifestyle modification was strongly emphasized as a critical component of achieving CHD prevention goals. In particular, dietary management was emphasized, including recommendation of a low saturated fat (less than 7% of caloric intake), low cholesterol (< 150 mg/day), mainly plant-based diet with calorie restrictions for overweight/obese persons. Stress management and coping skills along with physical activity also was emphasized, including recommendations of a regular exercise regimen (≥ 30 minutes of moderate intensity on most days). Cigarette smokers were encouraged to join a stop smoking program that may include use of the nicotine patch or other medications. Additionally, nicotine replacement pharmacotherapies were prescribed, when appropriate, to current smokers. Long-term adherence to these strategies and to medication therapies was stressed and evaluated at each appointment.

#### Delayed intervention

For the first 15 months following randomization, Delayed Intervention patients were expected to continue receiving on-going care from their PCPs. They received a folder at the conclusion of the baseline visit including handouts from the American Heart Association containing basic information about cardiovascular disease and a risk factor description sheet listing their biophysical measurements recorded at the baseline appointment as well as the ideal values for each measurement. They were told that they would be contacted by phone at 7 months and would begin case management intervention at 15 months. In addition, all PCPs received a letter outlining the CHD risk reduction goals recommended in the latest national guidelines.

### Statistical analysis and hypothesis testing

The primary hypothesis of the trial is that Immediate Intervention participants will experience greater reductions in 10-year CHD risk based on Framingham risk probability (primary outcome) than will Delayed Intervention participants. To test the primary hypothesis, we will compare Immediate Intervention participants relative to Delayed Intervention participants in a random-effects regression using SAS PROC GLIMMIX. The dependent variable will be the standardized Framingham risk score at 15-month follow-up. Initially, covariates will be limited to the baseline risk score and intervention status. So we will model participant i (i = 1, 2,..., n_jk_) under the care of physician j within clinic k as:

(1)     Risk_1ijk _= b_0 _+ b_1_Risk_0ijk _+ b_2_Int_ijk _+ α_j _+β_k _+ e_ijk_

where **Risk_1ijk _**is the standardized risk score for the i-th participant at 15 months (time 1) cared for by physician j in clinic k, **Risk_0ijk _**is the risk score at baseline (time 0), **Int_ijk _**is the intervention vs. control status of the i-th participant in the same clinic and under the same physician, **b_0 _**is a constant term, **b_1 _**is the coefficient associated with impact of baseline risk, and **b_2 _**is the coefficient associated with the impact of the intervention. **α_j _**represents the random effect caused by physician j and **β_k _**is the random clinic effect. The error term **e_ijk _**follows normal distribution, N(0, σ^2^). The analysis will be conducted on an intention-to-treat basis. The risk scores of participants lost to follow-up will be set to the baseline or interim values. Alternative methods for handling missing data, such as multiple imputation, may be used if appropriate. Our primary hypothesis will be confirmed if the coefficient associated with the intervention (**b_2_**) is significantly less than 0, implying that the intervention decreased CHD risk scores independent of the baseline level.

In addition, we will evaluate a number of secondary topics including: a) baseline differences in CHD prevention practices, b) moderators and mediators of the intervention effect, c) durability of the intervention effect, and d) cost effectiveness of the intervention. For example, some of the secondary hypotheses we will be testing include a) at baseline, CHD prevention practices within the SMMC fell significantly short of attaining guideline-based goals for a range of risk factors; b) at baseline, adherence to prevention guidelines varied directly by SES with adherence being least likely among participants of the lowest SES; c) implementation of the intervention had a greater impact on participants of lower SES thus resulting in a reduction on the magnitude of socioeconomic disparities in CHD prevention; d) changes in patient dietary and exercise habits were the largest mediators of the impact of the intervention; and e) the change in risk factors attributable to intervention was sufficient to achieve reasonable cost-effectiveness relative to other medical therapies.

In the current manuscript, we presented comparisons of baseline characteristics between the two study arms. All statistical analyses were performed in SAS for Windows (SAS Institute, Cary, NC). Frequency distributions, percentages in each group of categorical variables, and means and quartiles for continuous variables were generated for both intervention groups. Student's *t *tests were performed on continuous variables and χ^2 ^tests on categorical variables to assess comparability between the intervention groups at baseline. Statistical significance was set at *p *< 0.05 (two-tailed).

We will add appropriate covariates into equation (1) to perform the testing of the secondary hypotheses related to moderators and mediators of the intervention effect. For example, these covariates may include SES, change in caloric intake, and change in physical activity. The cost-effectiveness analysis will include: measurement of costs, measurement of changes in quality-adjusted life years (QALYs), and calculation of a cost-effectiveness ratio with an appropriate confidence region. The cost of implementing HTH will be estimated based on the cost of HTH staff time whereas the cost of implementing usual care will be derived from SMMC administrative records. We will estimate a statistical model of QALY changes based on the change in risk of death and, among survivors, the reduction in quality of life due to non-fatal events, which will be approximated using the collected health care utilization data. Using the cost and QALY figures we will estimate an incremental cost-effectiveness ratio, representing the cost per QALY due to the intervention, and a 95% confidence region surrounding the cost-effectiveness ratio using a bootstrapping method. Sensitivity analyses will be performed by varying the underlying model assumptions.

## Results

We achieved equal distributions of the demographic, biophysical and lifestyle characteristics between the two intervention groups. Each of these categories is reviewed below, with an emphasis on the aggregate characteristic of the entire population.

### Demographic characteristics (Table 2)

**Table 2:** Demographic characteristics of HTH participants relative to San Mateo County and the U.S. population^1^

		**HTH**^2^	**San Mateo County**	**U.S.**
**Mean Age in years (SE)**		55.7 (0.5)	53.7 (0.5)	54.3 (0.2)
**Sex **(%)	Male	34.6	47.8	47.4
	Female	65.4	52.2	52.6
**Ethnicity **(%)	Hispanic or Latino	62.8	14.9	10.2
	Non-Hispanic White	15.5	59.0	73.7
	African American	9.5	0.8	10.5
	Asian/Pacific Islander	12.2	22.9	4.2
	Other	0	2.4	1.4
**Education **(%)	< High School	61.3	16.1	15.2
	High School Graduate	15.9	15.3	32.3
	Some College	13.3	25.5	25.1
	College Graduate	8.9	43.1	27.3
**Marital Status **(%)	Never Married	16.5	14.6	9.9
	Married/Living with a Partner	39.3	62.0	64.1
	Separated	18.8	7.1	4.2
	Divorced	12.4	7.1	13.0
	Widowed	9.6	9.2	8.8
**Employment Status **(%)	Employed	38.2	63.5	61.3
	Unemployed	26.2	3.6	2.2
	Disabled or Otherwise Not in Labor Force	18.8	7.7	22.7
	Retired	13.9	25.1	13.8

^1^Estimates for San Mateo County and U.S. populations are based on the January 2006 Current Population Survey of adults between the ages of 35 and 85.

^2^The percentages for education, marital status and employment status do not add up to 100% because of missing data.

The mean age of HTH participants at baseline was 56 years (range 31 to 85 years). One participant whose age was 31 years at enrollment was randomized because of an incorrect date of birth, which was later rectified. We had expected to recruit an ethnically diverse population, including Latinos/Hispanics (55%) and other minorities (25%). The final sample consisted of 63% Latinos/Hispanics, 12% Asians and Pacific islanders, and 10% African Americans. In addition, 65% of the participants were female, 61% had less than a high school education, and 62% were not employed at the time due to unemployment, disability or retirement. This demographic profile differs from that of San Mateo County and of the U.S. as being more ethnically diverse and socioeconomically disadvantaged.

We also examined the distribution of participants by gender and ethnicity (Latinos/Hispanics vs. others) across the four study sites. The distribution of gender was comparable among clinics with women accounting for 59% of the participants in the South San Francisco clinic (total n = 100), 62% in the Menlo Park clinic (109), and 70% in the Redwood City (127) and Daly City clinics (83). The distribution of ethnicity varied across clinics. Eighty-seven percent of participants from the Redwood City clinic were Hispanic, accounting for 42% of all Hispanics in the entire sample. In addition, 78% of all blacks in the sample were from the Menlo Park clinic.

### Biophysical and lifestyle factors (Figure 2 and Table 3)

**Table 3:** Descriptive statistics for biophysical and lifestyle factors by study group^1^

	**Immediate Intervention **(n = 212)		**Delayed Intervention **(n = 207)	
	Mean (95% CI)	Median (25th, 75th)	Mean (95% CI)	Median (25th, 75th)
10-year CHD Risk (%)	15 (13 16)	11 (7 20)	15 (14 16)	11 (8 21)
BMI (kg/m^2^)	34.5 (33.7 35.3)	33.5 (29.1 38.6)	34.4 (33.5 35.4)	32.9 (29.0 37.8)
LDL-C (mg/dL)	104 (100 108)	102 (81 122)	104 (100 108)	103 (80 126)
HDL-C (mg/dL)	45 (44 46)	44 (37 52)	46 (45 48)	46 (38 54)
TC:HDL	4.4 (4.3 4.6)	4.2 (3.5 5.1)	4.5 (4.3 4.7)	4.0 (3.5 5.0)
TG (mg/dL)	197 (185 209)	177 (128 240)	204 (191 216)	174 (134 246)
TG:HDL	4.8 (4.3 5.2)	4.0 (2.8 5.9)	4.9 (4.4 5.4)	4.0 (2.7 5.8)
HbA1c	6.9 (6.7 7.1)	6.5 (5.5 7.9)	6.8 (6.7 7.0)	6.4 (5.7 7.7)
SBP (mmHg)	133 (130 135)	130 (120 144)	135 (133 137)	138 (120 146)
DBP (mmHg)	80 (79 81)	80 (70 88)	79 (78 81)	80 (70 86)
Fruits and Vegetables (#/day)	3.4 (3.3 3.6)	3.5 (2.7 4.2)	3.4 (3.3 3.6)	3.4 (2.4 4.3)
High Fat Foods (#/day)	3.5 (3.3 3.7)	3.3 (2.5 4.3)	3.7 (3.5 3.9)	3.5 (2.7 4.5)
Moderate and Vigorous Physical Activity (minutes/day)^2^	25 (18 33)	12 (0 30)	28 (20 37)	12 (0 34)

^1^None of the measures differed significantly by intervention groups at two-tailed P < .05.

^2^Calculated for participants with complete physical activity data (immediate intervention n = 141; delayed intervention n = 148). Abbreviations: BMI body mass index; LDL-C low density lipoprotein cholesterol; HDL-C high density lipoprotein cholesterol; TC total cholesterol; TG triglyceride; HbA1c hemoglobin A1c; SBP systolic blood pressure; DBP diastolic blood pressure.

**Figure 2:**
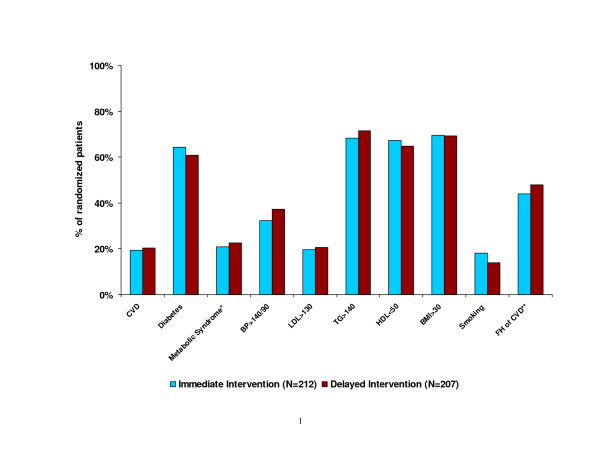
Proportion of randomized participants with CVD and CVD risk factors. *In the absence of diabetes. **FH: family history of cardiovascular disease

Twenty percent of participants in both the Immediate Intervention and Delayed Intervention groups reported having a prior CVD event. Sixty-three percent of participants had been diagnosed with diabetes, and an additional 22% had metabolic syndrome according to the Adult Treatment Panel III definition[23]. The average 10-year CHD risk was 15% (95% confidence interval [CI]: 13–16%) among HTH participants, with a median risk of 11% (interquartile range: 7–20%). Nearly 70% of participants in either group had a BMI > 30 kg/m^2^, with the mean BMI of 34.5 kg/m^2^. LDL-C levels averaged 104 mg/dL (95% CI: 100–108 mg/dL) with an interquartile range of 81 to 122 mg/dL. Depressed HDL-C, elevated TC:HDL ratio, elevated TG, and elevated SBP were common among participants. In particular, three-quarters of the participants had TG levels over 130 mg/dL or a TG:HDL ratio over 3.0, both suggesting insulin resistance [35]. In addition, 16% of participants self-identified as current smokers, and 45% had a family history of CHD or stroke. On average, HTH participants reportedly consumed 3.4 servings of fruits and vegetables per day, whereas their daily consumption of high-fat foods approached 4 servings. These participants also reported a daily average of 26 minutes of moderate- or vigorous-intensity physical activity.

We observed that several significant differences in biophysical and lifestyle factors by sex and ethnicity (Table 4). Women had lower 10-year risk for CHD than men (13% vs. 18%; p < 0.0001). When we removed the impact of gender on risk by calculating 10-year CHD risk for women using the male algorithm and vice versa, however, we found comparable levels of risk factor burden between two genders. Other gender differences included higher BMI and HDL-C levels, lower TC:HDL and TG:HDL ratios, and less physical activity in women compared with men. Compared with non-Hispanics, Hispanics had higher values of TC:HDL ratio, TG, and number of minutes of moderate- or vigorous-intensity physical activity.

**Table 4:** Differences in biophysical and lifestyle factors by gender and ethnicity^1^

	**Male **N = 145	**Female **N = 274	**Latino/Hispanic **N = 263	**Non-Latino/Hispanic **N = 156
Age in years	55.2 (0.8)	55.8 (0.6)	55.2 (0.6)	56.2 (0.7)
10-year CHD Risk	18 (1.0)^a^	13 (0.5)^b^	14 (0.6)	16 (0.9)
BMI (kg/m^2^)	33.2 (0.7)^a^	35.2 (0.5)^b^	34.1 (0.4)	35.1 (0.7)
LDL-C (mg/dL)	104 (3.0)	104 (2.0)	106 (2.1)	101 (2.7)
HDL-C (mg/dL)	41 (1.0)^a^	48 (0.7)^b^	45 (0.7)	47 (1.0)
TC:HDL	4.8 (0.2)^a^	4.2 (0.1)^b^	4.6 (0.1)^a^	4.2 (0.1)^b^
TG (mg/dL)	199 (9.8)	201 (5.9)	210 (6.6)^a^	183 (8.0)^b^
TG:HDL	5.4 (0.3)^a^	4.6 (0.2)^b^	5.2 (0.2)^a^	4.2 (0.2)^b^
HbA1c	6.9 (0.1)	6.8 (0.1)	6.8 (0.1)	6.9 (0.1)
SBP (mmHg)	133 (1.7)	134 (1.2)	133 (1.2)	135 (1.7)
DBP (mmHg)	81 (0.9)	79 (0.6)	79 (0.6)	80 (1.0)
Fruits and Vegetables (#/day)	3.3 (0.1)	3.5 (0.1)	3.5 (0.1)	3.3 (0.1)
High Fat Foods (#/day)	3.6 (0.1)	3.6 (0.1)	3.5 (0.1)	3.8 (0.1)
Moderate and Vigorous Physical Activity (minutes/day)^2^	35 (7.3)^a^	23 (2.4)^b^	32 (3.9)^a^	17 (3.4)^b^

^1^Data shown are mean and (SE).

^2^Calculated for participants with complete physical activity data (male n = 96; female n = 201; Hispanic n = 199; non-Hispanic n = 107).

^a, b^Different superscripts denote significant differences by gender or ethnicity at two-tailed P < .05.

### Baseline medical therapies (Figure 3)

**Figure 3:**
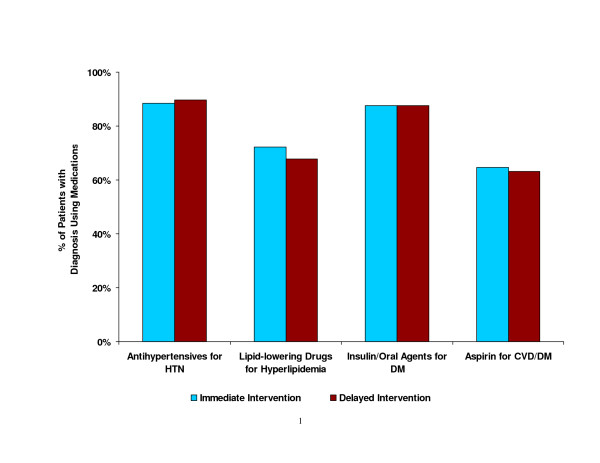
Proportion of randomized participants with specific diagnoses who were prescribed appropriate medication at baseline.

Prior to randomization, a large proportion of participants were taking medications for specific medical conditions. Eighty-nine percent of participants who had been diagnosed with hypertension received prescriptions for antihypertensive medications, and 69% of those with hyperlipidemia were prescribed lipid-lowering medications. Insulin or oral hypoglycemic agents were prescribed among 88% of participants with diabetes mellitus. Also, 64% of those with CVD or diabetes were taking aspirin. The proportion being treated at baseline for the selected conditions did not differ by sex and ethnicity.

## Discussion

As expected, the randomization process in the HTH effectively achieved an essentially equal distribution of socio-demographic, clinic and lifestyle characteristics between the two intervention groups. The lack of statistically and clinically significant differences on major potential confounders provides strong assurance for the internal validity of the clinical trial. The HTH sample is unique in its high composition of Latinos/Hispanics (62%) and other ethnic minorities (22%), persons with low educational attainment (61%), and persons without employment (62%). These population groups are clearly labeled in the literature as priority populations disproportionately affected by CVD and who are more likely to receive inferior CVD care [1,4,5]. The HTH sample exceeded our proposed target of enrollment for women (50%) and Latinos/Hispanics (55%). In addition, our sample consisted of 12% Asians and Pacific Islanders (target: 16%) and 10% African Americans (target: 10%).

By design, major cardiovascular risk factors are highly prevalent among HTH participants. 10-year CHD risk averaged 18% in men and 13% in women despite a modest LDL-C level and a high on-treatment percentage at baseline. This should not be surprising given that 63% of participants were diagnosed with diabetes, and an additional 22% with metabolic syndrome. In addition, many participants had depressed HDL-C levels and elevated values of TC:HDL ratio, TG, TG:HDL ratio, and blood pressure. Furthermore, nearly 70% of participants were obese, 45% had a family history of CHD or stroke, and 16% were current smokers.

A high proportion of the participants in our study were female (65%) although males are likely to be at higher risk for CAD. This reflects the overall higher usage of outpatient health care by women compared to men, as well as the greater availability of women for appointments during daytime working hours. To increase participation of men in future trials, setting aside evening clinic hours would enable men to come to appointments after their workday. In addition, case finding through women may lead to participation of their husbands although this would impose a more complex analytic design to account for the involvement of multiple household members in the same study.

The cardiovascular health profile of the HTH cohort strongly suggests a need for intensive cardiovascular risk reduction interventions, particularly lifestyle risk factor interventions. The interrelatedness of cardiovascular risk factors demands an integrated approach to management. However, the current US health care system lacks the capability of providing effective and cost-conscious CVD risk reduction interventions, particularly for ethnic minorities and low-SES populations [4-6]. Chronic disease management exerts tremendous time demands on PCPs [3,36] such that achieving guideline-accordant practice is unlikely unless physicians work as part of a health care team in which there is efficient division of labor. Case management provides an excellent team-approach model for integrating multiple risk reduction into practice that strives to meet nationally established goals for CVD risk reduction. Compared to usual care, case management has been shown to improve the delivery of care as well as resulting cardiovascular outcomes among predominantly white, high-risk patients [7-10]. Data are only beginning to accumulate with regard to the effectiveness of case management among ethnic minorities [11].

The HTH case management program is based on a model that has evolved through several previous clinical trials [7,8]. The model provides a systematic approach to the comprehensive, individualized and intensive management of cardiovascular risk in at-risk patients. It is based on the premise that cardiovascular risk reduction is synergistic and that CVD prevention and management is most successful when lifestyle interventions are integrated with appropriate medical therapies. At the core of the model is a team of nurses and dietitians (case managers) capable of treating hypertension, dyslipidemia, diabetes, obesity, physical inactivity, and smoking cessation. Case managers provide long-term counseling based on clinical status, risk level, interest in and readiness for change, and personal resources. Case managers' activities are integrated with the activities of the patient's PCP. Case management goals are modeled on latest practice guidelines.

To conclude, baseline characteristics of HTH participants suggest that we have recruited an appropriate cohort in which to implement a case management approach and test its efficacy and cost-effectiveness. Due to its unique composition of ethnic minorities and persons of low-SES, the HTH will enrich the U.S. literature regarding better strategies for CVD prevention among these priority population groups and aid in guiding future practice that will reduce health disparities. The HTH experience also will be of considerable learning value for health care systems in Canada, the United Kingdom, and other countries that have shown increasing interest in the clinical utility of the case management model [37-39]. Our experience to date has led to success in a number of areas that are likely to translate into final success of the project. Among these: 1) developing a mutually supportive and productive collaboration with SMMC; 2) developing the HTH model both in terms of its intellectual and evidence-based foundation, as well as all its logistical elements; 3) successfully recruiting slightly more than the expected number of patients; 4) recruiting and retaining a cohesive staff of providers and researchers; 5) eliciting a favorable response from San Mateo County regarding the intervention and plans to disseminate the intervention to additional clinical sites as a County-run program.
